# Acknowledgement to reviewers

**DOI:** 10.1530/RAF-6-A1

**Published:** 2025-02-26

The editorial board wishes to thank the following individuals who have helped to review manuscripts for *Reproduction & Fertility* during 2024. Their assistance is gratefully acknowledged.

A O Afferri

A Ahmad

M Almeida

A Anderson

A Andonissamy

A Anger

A Atkinson

A F Basini

A Belchamber

A Bernard

A M Blitek

M Bloom

A Boafo

A Bromfield

A Campbell

A Capalbo

G Carrillo

B Chan

B R Chung

B J Delgouffe

C Dhar

Z Druitt

C Duan

C Duncan

C Edagha

D El-Mazny

D Ennis

D Fatum

H Foot

A Fraire-Zamora

D Gardner

M F Gasparini

M Gentek

Y George

E Gerrits

E Ghanami Gashti

F Ghersevich

F Gholiof

F Gonzales

T Gubbels

G Gubbels

G Gullo

G Hawdon

M Hicks

G Hirsch

G Hobbs

G Houston

H Huang

C Huang

B Hull

H Ibrahim

H Inal

H Inhorn

H Jagannathan

I Jahangiri

I A Ježek

I Johnston

J Ka

T Kamaraj

J Kamble

N Kamrul-Hasan

J Karunakaran

J Khaw

J Ko

J Kotarska

J Lam

J Lea

J Lee

J Leonardi

J Li

J Liperis

J Loman

K Lopes

E Mardon

N Marron

K Massarotti

K McAllister

K Mehdinejadiani

K K Moldenhauer

K Moll

L Morgan

M Morrell

L Murray

L Novero

M Oliveira

L Oliver

M Ombelet

M Opsomer

M Ortega

M Osamu

M Ozgu-Erdinc

M E Palomba

M Papadopoulou

M L Patel

M Pavani

M Pedrana

J Pereira

S Peters

M Poulat

M Qasemi

M Quinton

M Ralapanawe

M Relovska

N Robertson

N Rosales-Torres

N Rosario

N Rosen

N Rossouw

N Ryan

H Sah

Y Salami

P T Sallam

S Saunders

A Seo

R Serdarogullari

R Serour

R Sheibak

R Shokoohi

R Sims

R Siqueira

R Siristatidis

S Soler

S Stathatos

S Stevens Brentjens

A Subiran

S Sun

S A Terraube

S C Thabet

M S Tremellen

S Tyagi

S Ugwu

S Uloho

S T Uraji

Y Veleva

T Walker

P Warkiani

V Watkins

V Wilkinson

W C Woolner

W Yamashiro

Z Zadeh

Z Zhongwei

